# Effects of topical timolol for the prevention of radiation-induced dermatitis in breast cancer: a pilot triple-blind, placebo-controlled trial

**DOI:** 10.1186/s12885-022-10064-x

**Published:** 2022-10-20

**Authors:** Mohsen Nabi-Meybodi, Adeleh Sahebnasagh, Zahra Hakimi, Masoud Shabani, Ali Asghar Shakeri, Fatemeh Saghafi

**Affiliations:** 1 Department of Pharmaceutics, School of Pharmacy, Shahid Sadoughi University of Medical Sciences and Health Services, Yazd, Iran; 2 Clinical Research Center, Department of Internal Medicine, School of Medicine, Faculty of Medicine, North Khorasan University of Medical Sciences, Bojnurd, Iran; 3 Pharmaceutical Sciences Research Center, School of Pharmacy, Shahid Sadoughi University of Medical Sciences and Health Services, Yazd, Iran; 4 Department of Radiooncology, School of Medicine, Shahid Sadoughi University of Medical Sciences and Health Services, Yazd, Iran; 5 Department of Clinical Pharmacy, School of Pharmacy, Shahid Sadoughi University of Medical Sciences and Health Services, Yazd, Iran; 6 Shahid Sadoughi University of Medical Sciences, Department of Clinical Pharmacy, Faculty of Pharmacy, Shohadaye gomnam Blvd, Yazd Province Yazd, Iran

**Keywords:** Timolol, Radiodermatitis, Breast cancer, Clinical trial

## Abstract

**Introduction:**

Radiation therapy is one of the standard methods in the treatment of breast cancer. Radiotherapy-induced dermatitis (RID) is a common complication of radiotherapy (RT) resulting in less tolerance in RT and even discontinuation of treatment. Timolol is a β-adrenergic receptor antagonist that presents the best wound healing effects on both chronic and incurable wound healing. Topical forms of timolol could be effective in the prevention of RID due to the role of β-adrenergic receptors in skin cells and keratinocyte migration, as well as the anti-inflammatory effect of timolol. However, no placebo-controlled randomized trial is available to confirm its role. The current trial aimed to evaluate the efficacy of topical timolol 0.5% (w/w) on the RID severity and patients' quality of life (QOL)**.**

**Method:**

Patients aged older than 18 years with positive histology confirmed the diagnosis of invasive and localized breast cancer were included. Patients were randomized based on the random number table to receive each of the interventions of timolol 0.5% (w/w) or placebo topical gels from the first day of initiation of RT and for 6 weeks, a thin layer of gel twice daily. Patients were asked to use a thin layer of gel for at least two hours before and after radiation therapy. Primary outcomes were acute radiation dermatitis (ARD) grade using Radiation Therapy Oncology Group and the European Organization for Research and Treatment of Cancer (RTOG/EORTC) scale and severity of desquamation based on Common Terminology Criteria for Adverse Events (CTCAE), version 5.0. Secondary outcomes were QOL based on Skindex16 (SD-16), maximum grade of ARD, and time of initial RD occurrence.

**Results:**

A total of 64 female patients with an age range of 33 to 79 years were included. The means (SD) of age were 53.88 (11.02) and 54.88 (12.48) in the control and timolol groups, respectively. Considering the RTOG/EORTC and CTCAE scores the difference between groups was insignificant (*P-Value* = 0.182 and *P-Value* = 0.182, respectively). In addition, the mean (SD) of time of initial RID occurrence in placebo and timolol groups were 4.09 (0.588) and 4.53 (0.983) weeks, respectively (*P-Value* = 0.035). The maximum grade of RID over time was significantly lower in the timolol group. During the study period, 75.0% of patients in placebo groups had grade 2 of ARD while in the timolol group it was 31.3% (*P-Value* = 0.002). QoL was not significantly different between groups (*P-Value* = 0.148).

**Conclusion:**

Although the topical formulation of timolol, 0.5% (w/w), was found to reduce the average maximum grade of ARD and increase the mean (SD) time of initial RID occurrence, it showed no effect on ARD, severity, and QOL. However, future clinical trials should be performed to assess timolol gel formulation in larger study populations.

**Trial registration:**

https://irct.ir/ IRCT20190810044500N11 (17/03/2021).

## Background

Cancer is one of the leading causes of mortality worldwide, and cancer treatment options include chemotherapy, radiotherapy, surgery, and hormone therapy [1]. RT is a treatment based on the use of high-energy waves or radioactive particles to damage tumor cells to attenuate their growth. This modality has been effectively used for cancer treatment in more than 100 years [2]. Approximately 75% of cancer patients receive radiation therapy as a part of their treatment [3].

RT is one of the standard protocol with a high success rate for the treatment of breast cancer to reduce the risk of recurrence and death [4, 5]. The goal of RT is to destroy tumor cells with minimal damage to normal tissue. However, normal cells may be damaged when exposed to radiation. Exposure to ionizing radiation produces free radicals that can damage cellular DNA, change proteins, carbohydrates, and lipids, release the inflammatory cytokines and structural damage to the skin. Normally, natural tissues have a high capacity for self-repair but an imbalance between tissue damage and repair occurs when cells are exposed to repeated radiation [3].

RID occurs in 95% of patients receiving RT during their treatment [6]. The skin cells located in close vicinity to the tumor cells receive large amounts of radiation, causing several complications such as redness, dry and wet desquamation, and tissue necrosis [7, 8]. Wet desquamation may lead to the perception of a severe pain around the skin folds [4, 9]. One common complication of RT is radiation induced primary and delayed dermatitis. Primary reactions include erythema, dry skin, moist desquamation, and sometimes wound. The most common symptoms of delayed dermatitis are fragile or thin skin, fibrosis, acanthosis, skin pigmentation, atrophy, telangiectasia, sensitivity to trauma, neuropathy, and cutaneous neoplasms [5, 10]. The occurrence of these complications in patients lead to discomfort, limited daily activities, and even stop radiotherapy, which negatively affects the cancer treatment [5, 11]. Symptoms usually appear 10–14 days following the initiation of treatment and carry on for 2 to 4 weeks during RT [3, 12]. The severity of dermatitis depends on the dose per fraction, total dose, radiation quality, radiation method, pre-chemotherapy, and skin type [13, 14]. Notably, the patient and radiotherapy characteristics also affect the frequency and severity of skin reactions [4, 15].

Previous studies evaluated the effect of various topical formulations in RID, such as aloe vera, anionic phospholipids and hyaluronic acid based formulation, and corticosteroids [16–20]. However, until now, there is no standard measure for prevention of RID in patients with breast cancer.

Oral and topical formulations of timolol, a nonspecific β-receptor antagonist, are indicated in the management of glaucoma, myocardial infarction, hypertension, and prophylaxis of migraine headache. β_2_ adrenergic receptor is an important regulator of wound regeneration. Previous experimental and clinical studies have shown that this receptor plays an important role in skin cell migration and proliferation. β_2_ adrenergic receptor also modulates re-epithelialization, angiogenesis, and inflammatory responses during processes of wound healing [21, 22]. Direct migration of keratinocytes is critical for wound re-epithelialization. β-adrenergic receptor signaling system plays a key role in epidermal wound physiology. Activation of β_2_ receptors delays the regeneration of the epidermal barrier, while blockage of this receptor promotes the regeneration of these boundaries. Thus, it is presumed that blocking the β adrenergic receptors of keratinocyte enhances the rate of their migration and accelerated the process of re-epithelialization of wound [23]. The beneficial effects of topical timolol 0.5% have been exhibited previously in the management of chronic foot ulcers [24], surgical scars [25], chronic hand eczema [26], trauma wounds, vascular complications [27], and chronic wounds [12, 28].

Catecholamines are endogenous agonists for adrenergic receptors, and epinephrine has the highest specificity for β adrenergic receptors. Epinephrine prevents the migration of keratinocytes through β adrenergic receptor. Keratinocytes contain enzymes that are essential for synthesis of epinephrine. Environmental stress (e.g., UVB radiation and heat damage) regulates the expression of cyclic adenosine monophosphate (cAMP) and β_2_ receptors in keratinocytes [23, 29–31]. The expression of Phenylethanolamine N-methyltransferase enzyme is increased in wound site through the destructive effects of radiation and heat, which subsequently promotes the production of epinephrine and delays wound healing processes. Thereby, the topical timolol, as an antagonist of β adrenergic receptors, could be a potential candidate in the enhancement of wound healing process by preventing the binding of epinephrine to β_2_ receptors [12, 32].

Exposure to ionizing radiation results in the production of free radicals and release of inflammatory cytokines which subsequently damages the keratinocytes and vascular endothelial cells, all contributes in the structural damage into the epidermis and dermis [33]. On the other hand, the positive therapeutic effects of timolol are attributed to the antioxidant activity of this drug on the entire cell [34]. The clinical studies have shown that timolol protects the endothelial cells from oxidative stress with its potent antioxidant activity [35]. β adrenergic receptor antagonists could exhibit anti-inflammatory action through reducing lymphocyte proliferation, circulating natural killer cells, and T lymphocytes [27]. Therefore, Timolol, as an β-adrenergic receptor antagonist, with its antioxidant, anti-inflammatory and wound-healing properties, can interfere with the underlying pathogenesis of RID and damage to the irradiated epidermis and dermis. Despite the introduction of numerous treatment options in recent years, no effective treatment is available for prevention of RID. Considering the underlying pathogenesis of RID and the mechanism of actions of timolol, this study aimed to determine the role of this β-adrenergic antagonist in the prevention of RID. To our knowledge, this is the first clinical trial of timolol in this bothersome complication of RT in breast cancer patients.

## Methods

### Ethics considerations

The study protocol was approved by the Ethics Committee of Shahid Sadoughi University of Medical Sciences (IR.SSU.MEDICINE.REC.1399.058) and registered in the Iranian Registry of Clinical Trials (IRCT20190810044500N11). Informed consent was obtained from all subjects or their legal guardians. All experiments were performed in accordance with relevant guidelines and regulations.

### Materials

Timolol maleate as active pharmaceutical ingredient (API (was purchased from Sina darou Laboratories (Tehran, Iran). Polyethylene glycol 4000 and propylene glycol 99.0% were provided by Samchun Chemicals (Gyeonggi-do, Korea). Poly (1-carboxyethylene) or carbopol® 934 as a thickener were purchased from Serva FeinBiochemica (Heidelberg, Germany). Furthermore, triethanolamine as pH adjusters was supplied by Merck (Darmstadt, Germany).

### Topical gel preparation

The topical gels were prepared in the pharmaceutics laboratory of a pharmacy school. For preparation of 50 g topical timolol gel 0.5% (w/w), 200 mg carbopol® 934 was added slowly to 44.46 g of stirring phosphate buffer for 24 h. Then, 0.34 g timolol maleate powder was dissolved in 5 g propylene glycol. In the next step, two prepared solutions were mixed. Triethylamine was added until the pH was 7. Placebo gel was prepared with the same materials except timolol. Finally, both topical preparations were packed in similar 50 g aluminum collapsible tubes. The stability test was performed in terms of its organoleptic properties such as clarity, consistency, homogeneity, and spreadability. The prepared gels were stable in the refrigerator (4 °C) for at least one week. Then, the tubes were labeled A or B by the principal investigator.

### Participants

Patients aged 18 years or older with a pathologic diagnosis of breast cancer, receiving a radiation dose of maximum 60 Gy in 200 cGy fractions who were referred to a medical university-affiliated radiotherapy center were evaluated for eligibility. Patients with known allergy or contraindication of β-blockers, unwillingness to sign an informed consent, inflammatory metastatic carcinoma, concomitant use of nonsteroidal anti‐inflammatory drugs (NSAIDs), corticosteroids, and other immunosuppressive or antioxidant medications, chronic skin or connective tissue diseases were not included to the study. Exclusion criteria were lack of cooperation to continue treatment, and improper use of the study gel and poor compliance which was evaluated by eight-item Morisky Medication Adherence Scale (MMAS-8) [36, 37]. This tool applies a series of short behavioral questions geared in such a way to avoid “yes-saying” bias. The higher scores in this scale are in favor of more adherent. If the patients developed grade 3 dermatitis according to RTOG/EORTC and CTCAE criteria [3, 38], they would have been transitioned off the study medication and given standard of dermatologic care.

### Trial design and blinding

The patients, the radiation oncologists, and the investigator of clinical responses were all blinded to the intervention assignments throughout the study. The principal investigator, who was unaware of the interventions, gave A or B codes to each prepared formulation. After the accomplishment of the clinical phase of the study, the principal investigator decoded the topical formulations and assigned each one to the appropriate group.

Patients were randomized to receive each of the interventions of timolol 0.5% (w/w) or placebo topical gels. Radiation dose was 50–60 Gy in 200 cGy fractions given over 5 days per week. The skin examination was performed at the baseline to confirm no previous skin disease. Patients were asked to use a thin layer of gel twice a day for at least two hours before and after radiation therapy. Patients were recommended not to wear their clothes ten minutes after the topical applying the gel and do not wash the area until performing radiotherapy. Patient were also prohibited to use other topical and/or systemic agents for prophylaxis of dermatitis. During radiotherapy, all patients were given the necessary skin care recommendations according to the Multinational Association for Supportive Care in Cancer (MASCC) Skin Toxicity Study Group guideline to prevent acute skin reactions caused by radiotherapy (1). After the accomplishment of the clinical phase of the study, the principal investigator decoded the topical formulations and assigned each one to the appropriate group.

### Randomization

Randomization in a 1:1 ratio was used to ensure a balanced allocation of 64 eligible patients in the control and timolol groups. The random allocation sequence was generated using random allocation software (version 1). Thereby, the first eligible person was referred to as number 1, the second person as number 2, and so on until the 64^th^ patient. Next, using the software generated list, the patients received one of the interventions. To access allocation concealment, an examiner (who was not involved in the study) performed randomization.

### Outcomes

Demographic characteristics of the participants were recorded at baseline. Primary and secondary outcomes were evaluated at baseline, then weekly during RT, and finally 2 weeks after the termination of radiotherapy course. Primary outcome was the grade of ARD using each of RTOG/EORTC and CTCAE version 5.0. The severity of ARD was undertaken every week in accordance with the criteria of the RTOG/EORTC and the size and severity of skin ulceration was scored using the CTCAE (Table 1). Secondary outcomes were QOL based on Skindex16 (SD-16), maximum recorded grade of ARD during the study follow-up, and the time of initial RID occurrence.

**Table 1:** Acute radiation dermatitis corresponding to the RTOG/EORTC and CTCAE scoring criteria

	Grade					
	0	1	2	3	4	5
RTOG/EORTC	No skin rending, ulceration, inflammation or damage	Follicular, faint or dull erythema or dry desquamation	Moderate to brisk erythema, patchy moist desquamation mostly confined to skin folds and creases, moderate edema	Confluent, moist desquamation other than skin Folds (≤ 1.5 cm diameter), pitting edema	ulceration of full thickness dermis or skin necrosis, spontaneous bleeding from the involved site	Death
CTCAE (v5.0)	No dermatitis	Combined area of ulcers < 1 cm, faint erythema or dry desquamation	Combined area of ulcers 1—2 cm; moderate to brisk erythema, patchy moist desquamation, moderate edema	Combined area of ulcers > 2 cm; full-thickness skin loss involving damage to subcutaneous tissue that may extend down to fascia	life-threatening consequences, skin necrosis or ulceration of full thickness dermis, spontaneous bleeding from involved site, skin graft indicated	

*RTOG/EORTC* Radiation therapy oncology group and the european organization for research and treatment of cancer; *CTCAE* (v5.0) Common terminology criteria for adverse event (version 5)

### Sample size

The current pilot study was developed to calculate the sample size for a larger trial. Therefore, considering the rule of thumb for the pilot studies, at least 12 participants in each group would be an appropriate justification for sample size [39]. Considering low participation of the patients during COVID-19 pandemic, with the allowance of possible lost to follow-up during the study period, we allocated 32 patients to the control group and 32 patients into timolol 0.5% group.

Data from a previous randomized prospective trial was used for sample size calculation [3]. A total study size of at least 54 patients (2 × 27 patients per each group) using the following equation allowed for a power (1-β) of 85% at a significance level of 0.05 and ARD grade by RTOG/EORTC score ≥ 2 at weeks 1 to 6 for detecting a difference between two proportions (reduction in total clinical score) of at least 40% (30% vs 75%). The estimated sample size was increased to 32 per group to take account of potential attrition of 12%.

$$n=\frac{{\left(z\frac{\alpha }{2}+z\beta \right)}^{2}\left[{p}_{1}\left(1-{p}_{1}\right)+{p}_{2}\left(1-{p}_{2}\right)\right]}{{\left({p}_{1}-{p}_{2}\right)}^{2}}$$

### Statistical analysis

The Kolmogorov–Smirnov (KS) test was used for checking normality of the data. The quantitative and qualitative variables were reported as each of mean (SD)/median (IQR) and frequency (%), respectively. The distributed quantitative variables were compared between groups by using the Mann–Whitney U test. Moreover, repeated measurement was used to compare changes of variables at groups over time. Spearman’s rank correlation coefficient was used to evaluate the association between body mass index (BMI) and ARD. Data were analyzed using statistical package for social science (SPSS) software version 23.0 and *P*-values < 0.05 were considered statistically significant.

## Results

**Fig. 1:**
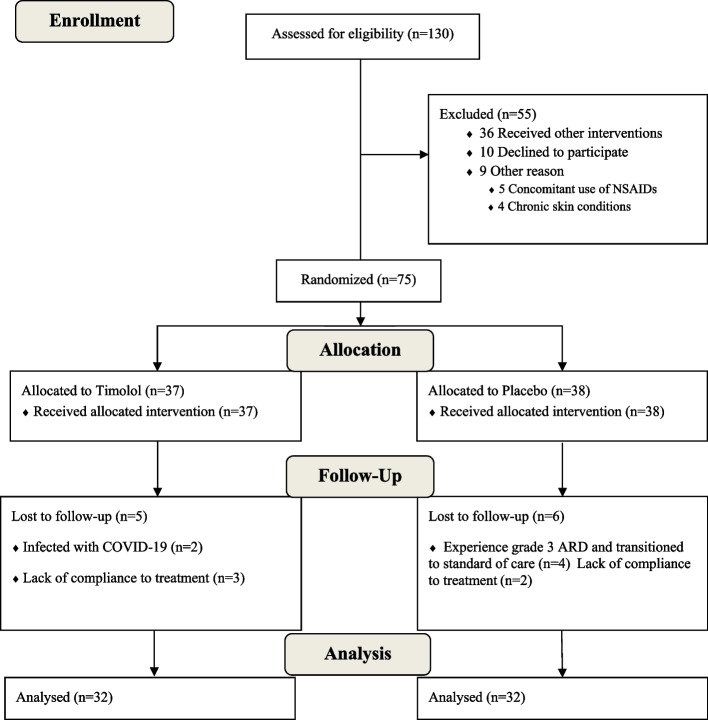
CONSORT flow diagram of Timolol 0.5% (w/w) vs placebo during study follow up. NSAIDs: Non-steroidal anti-inflammatory drugs; COVID-19: Coronavirus Disease of 2019; ARD: Acute Radiation Dermatitis

In this study, 130 new cases of breast cancer who were referred to a medical university affiliated radiotherapy center were screened. Fifty-five patients were excluded from the study, because of consuming other topical interventions, decline to participate, history of asthma or cardiovascular diseases, and previously known sensitivity to β-blockers. Eventually, 75 subjects were randomized to receive each of topical timolol 0.5% (w/w) (*N* = 37) or placebo gels (*N* = 38). In the placebo group, two patients were excluded, because of not using the topical preparations properly and four experienced grade 3 ARD. In the timolol group, three patients were excluded, because of not using the topical preparations properly and two contracted coronavirus disease 2019 (COVID-19). Sixty-four patients completed the study and have yielded data for analysis (Fig. 1). Demographic and baseline clinical characteristics of enrolled patients are given in Table 2.

**Table 2:** Patient demographic profile and baseline disease characteristics

Parameters	Groups		*P-Value*
	**Timolol**	**Placebo**	
	(*N* = 32)	(*N* = 32)	
Age, Mean (SD)	53.8 (11.0)	54.8 (12.4)	0.735
BMI, kg/m^2^	23.1 (2.9)	22.8 (3.3)	0.551
Coexisting conditions, N (%)			
Hypertension	4 (12.5)	3 (9.4	0.457
Diabetes mellitus	5 (15.6)	7 (21.9)	
Dyslipidemia	6 (18.7)	7 (21.9)	
Skin phototype, N (%)			
Type 1	0 (0.0)	0 (0.0)	0.143
Type 2	0 (0.0)	3 (9.4)	
Type 3	19 (59.4)	21 (65.6)	
Type 4	12 (37.5)	6 (18.7)	
Type 5	1 (3.1)	2 (6.3)	
Laterality, N (%)			
Right	10 (31.0)	11 12 (34.0)	0.224
Left	21(66.0)	17 (54.0)	
Both	1 (3.0)	4 (12.0)	
Type of breast surgery, N (%)			
BCS	28 (87.5)	29 (90.6)	0.782
MRM	4 (12.5)	3 (9.4)	
History of chemotherapy, N (%)			
Yes	14 (43.7)	13 (40.6)	0.800
Smoking status, N (%)			
Yes	0 (0.0)	1 (3.1)	0.332
Marital status, N (%)			
Single	2 (6.3)	2 (6.3)	1.000
Married	30 (93.7)	30 (93.7)	
Her2 status			
Positive	11(34.4)	14(43.7)	
Hormone receptor status			0.442
ER^+^/PR^+^	19 (59.4)	22 (68.7)	0.736
ER^+^/PR^−^	4 (12.5)	3 (9.4)	
ER^−^/PR^−^	9 (28.1)	7 (21.9)	

*N* Number, *SD* Standard deviation, *BCS* Breast-conserving surgery, *MRM* Modified radical mastectomy, *HER2* Human epidermal growth factor receptor 2, *ER* Estrogen receptor, *PR* Progesterone receptor. Independent-Samples T Test and Chi-squared test were used to compare these values

### Primary outcomes

The intention to treat analysis of the ARD grade by RTOG/EORTC and CTCAE scores showed a significant difference between timolol and placebo groups at weeks 4 to 6 (*P*-Value < 0.05), but not at the end of the first two weeks (Table 3). Moreover, the median of RTOG/EORTC and CTCAE scores were zero for all patients in both groups at baseline while the median increased to score 1 and 2 in the timolol and placebo groups, respectively, at the end of week 6 (Tables 3 and 4). There was also a statistically significant time effect (*P*-Value < 0.001), but the difference between the two groups in time × group interaction effect was not statistically significant (*P-Value* = 0.182).

**Table 3:** Primary and Secondary Outcomes over time during weeks 1 to 7 for Timolol and 4 Placebo groups

Outcome	Week	Groups		Betwee n groups *P*-value	Effect of time *P-Value*	Effect of Time × group *P-Value*	Overall *P*-value
		Timolol	Placebo				
		Median (IQR)					
ARD using RTOG/E ORTC	1	0.0 (0.0)	0.0 (0.0)	1.00	< 0.001	0.341	0.182
	2	0.0 (0.0)	0.0 (0.0)	1.00			
	3	0.0 (0.0)	0.0 (0.0)	0.166			
	4	1.0 (1.0)	1.0 (1.0)	< 0.001			
	5	1.0 (0.0)	1.0 (0.0)	0.001			
	6	1.0 (1.0)	2.0 (1.0)	0.013			
	^1^Follow up	0.0 (1.0)	1.0 (0.0)	0.105			
ARD using CTCAE	1	0.0 (0.0)	0.0 (0.0)	1.00	< 0.001	0.341	0.182
	2	0.0 (0.0)	0.0 (0.0)	1.00			
	3	0.0 (0.0)	0.0 (0.0)	0.166			
	4	1.0 (1.0)	1.0 (1.0)	0.001			
	5	1.0 (0.0)	1.0 (0.0)	0.001			
	6	1.0 (1.0)	2.0 (1.0)	0.031			
	^1^Follow up	0.0 (1.0)	1.0 (0.0)	0.105			
QOL using Skindex16	1	0.0 (0.0)	0.0 (0.0)	1.00	< 0.001	0.007	0.148
	2	0.0 (0.0)	0.0 (0.0)	1.00			
	3	1.0 (2.0)	1.0 (2.0)	0.453			
	4	6.0 (7.0)	6.0 (4.0)	0.008			
	5	17.0 (3)	17.5 (0.0)	0.001			
	6	25.0 (14.0)	40.0 (5.0)	0.014			
	^1^Follow up	2.0 (13.0)	10.5 (7.0)	0.284			

*IQR* Interquartile range, *RTOG/EORTC* European organization for research and treatment of cancer, *CTCAE* Common terminology criteria for adverse events, *QOL* Quality of life, ^1^Follow up is referred to “within 2 weeks after the completion of radiation therapy”; General Linear Model and Mann–Whitney U test was used to compare these values

^*^Statistically significant (*P-value* < *0.05*)

**Table 4:** Maximum severity of ARD corresponding to the RTOG/EORTC score in included patients during the study follow-up visits^1^

ARD severity	**Timolol**	**Placebo**	***Overall P-Value***
		**N (%)**	
0	1 (3.1%)	0 (0.0%)	0.002
I	21 (65.6%)	8 (25.0%)	
II	10 (31.3%)	24 (75.0%)	
III	0 (0.0%)	(12.5)	

*N* Number, *ARD* Acute radiation dermatitis

^1^Follow up is referred to “within 2 weeks after the completion ofof ending the radiation therapy”

### Secondary outcomes

The maximum severity of ARD was lower with the timolol group compared to the placebo when treated prophylactically (*P*-Value = 0.002). Only 31.3% of patients receiving timolol experienced RTOG/EORTC grade II compared to 75.0% of patients receiving placebo. Furthermore, despite the fact that 31 (96.9%) patients in the timolol group experienced ARD at the end of the study, none of them suffered ARD more severe than Grade 2. While on the contrary, in placebo group, 40% of patients experienced Grade 2 and three patient experienced Grade 3 of ARD, which were excluded from the study. Furthermore, one participant in timolol group remained asymptomatic at the end of the study. The details of our findings are given in Table 4.

In terms of skin-related QOL, evaluated by the Skindex-16 (SD16) questionnaire, there were no differences between the two groups at weeks 1 to 3 (*P*-Value > 0.05). This value increased dramatically during weeks 4 to 6 and then started to fall gradually. However, the values of these changes at week 6 of RT were much higher for the placebo group compared with the intervention group (Table 2).

Furthermore, the mean (SD) time of incidence of ARD in placebo and timolol groups were 4.09 (0.588) and 4.53 (0.983) in weeks, respectively, which was statistically significant (*P*-Value = 0.035). In order to evaluate the association between BMI and ARD, spearman’s rank correlation coefficient was used. The results showed no significant association between BMI and ARD (spearman’s rank correlation coefficient = 0.017 and *P-value* = 0.895).

### Adverse effects

Mild adverse effects, sensed as the feeling of irritation was reported in all the 64 sites treated with each of timolol or placebo topical formulations. However, none of the patients discontinued the therapy because of the adverse effects. No reports of bradycardia or wheezing were reported in any of the patients who completed the treatment period.

## Discussion

Although the anti-inflammatory properties of timolol, the data on its’ radioprotective effects is limited. The present study was the first randomized, controlled clinical trial evaluating the efficacy and safety of timolol 0.5% (w/w) topical gels twice a day at least two hours before and after receiving RT in prevention of RID. The results of the present study demonstrated that timolol 0.5% (w/w) topical gel can significantly delay and decrease the incidence of ARD and its severity in breast cancer patients receiving RT compared with those receiving the placebo. Moreover, the maximum grade of RID over time was significantly diminished in timolol groups.

RID is the most common adverse effect of breast-cancer RT. During RT, around 95% of patients develop some degree of local inflammatory symptoms, such as erythema, dry or moist desquamation, edema, and ulcers. The severe presentations of radiodermatitis, e.g., moist desquamation, ulcers, and skin fibrosis, may necessitate discontinuation of the RT. This subsequently impair patients' QOL and negatively influence the outcomes of the patients. The pathogenesis of radiodermatitis is rather complex and comprises of radiation tissue injury followed by an inflammatory reaction. An erythematous skin reaction develops by an increased vascular permeability and vasodilation. This is followed by inflammatory responses [40].

Wound-healing is a well-organized and a complex process achieved through four distinct phases of hemostasis, inflammation, proliferation, and remodeling [41]. During the past two decades of research, the efficacy of various biological and chemical compounds such as antioxidants, cytoprotective factors, and vitamins have been investigated [42–44]. Yet, no proven modality is available for prevention of RID. Topical steroids such as mometasone 0.1% and hydrocortisone have been evaluated for their anti‐inflammatory properties [16, 45]. The results of the previous studies suggested that low dose of corticosteroids may be beneficial in reducing itching and irritation in patients with radiodermatitis. Moreover, steroids are contraindicated in the presence of infection as they could mask the signs and symptoms of infection and worsen it [16, 18, 45].

The first clues to the biological effect of β -adrenergic receptor in wound-healing process came from Donaldson study, revealing that β -adrenergic receptor agonists delay wound repairing in newt limbs [46]. Later studies confirmed that β adrenergic receptor antagonists promote wound re-epithelialization through blocking the β2 receptors within the skin layers [23, 47, 48]. The efficacy of β adrenergic receptor antagonists in promoting wound healing process was initially demonstrated by their systemic administration [49]. Despite limited clinical evidence to support the efficacy of topical timolol, Thomas et al. in a case–control study reported that topical application of 0.5% timolol solution along with antibiotics and dressings produced clinically significant reduction in ulcer area within 4 weeks [24]. Mohammadi et al., in a randomized double-blind clinical trial showed oral propranolol decreased healing time of superficial wounds and hospital stay period in hospitalized burn patients [47]. Furthermore, several case reports of illustration of topical timolol effects on acute and refractory chronic wounds healing have been published [12, 25, 27]. In addition, β adrenergic receptor antagonists could exhibit anti-inflammatory action through reducing lymphocyte proliferation, circulating natural killer cells, and T lymphocytes [27]. Although the main mechanisms for β adrenergic receptor antagonists is not known, the proposed mechanisms are as follows: accelerate re-epithelialization, reduce inflammatory response, increase fibroblast migration and angiogenesis, and enhance extracellular signal-related kinase phosphorylation [23].

The application of topical silver sulfadiazine in breast cancer patients referred for RT indicated that women in silver sulfadiazine encountered less sever ARD compared with patients in the control group [50]. The results of another trial revealed that topical administration of atorvastatin 1% significantly reduced severity of ARD compared with placebo [51]. The results of the current study have overall showed that topical administration of timolol 0.5% gel was superior to the placebo gel in the prevention of the ARD incidence and related symptoms.

Previously, compounds with similar anti-inflammatory and antioxidant properties have been used successfully for this complication. For instance, the anti-inflammatory and antioxidant activity of herbal products has been demonstrated in different experimental and clinical evidences [3, 20, 40]. Rafati et al. demonstrated that the topical administration of Nigella sativa 5% gel with anti-inflammatory and antioxidant properties delayed and decreased the severity of ARD and its related symptoms compared to the placebo (3). In this study, we observed that the topical application of timolol 0.5% to the radiation-exposed breast area can effectively prevent the occurrence of ARD.

Karbasforooshan et al. performed a randomized, double‐blind, clinical trial on 40 breast cancer women who were referred to receive RT. The eligible patients were randomly allocated to receive silymarin 1% gel or placebo once daily from the first day of radiotherapy for 5 weeks. The acute skin reactions were assessed according to RTOG/EORTC and CTCAE criteria. However, after 5 weeks of RT, only 9.8% of patients in silymarin group experienced Grade 2 radiodermatitis in comparison with 52% in placebo group. At the end of the RT, proportion of patients without RID was significantly higher in silymarin group (23.5% vs. 2%, p < 0.02). The current study found that 31.3% of participants in timolol group experienced Grade 2 radiodermatitis in comparison with 75.0% in placebo group at study termination [40].

Although the results of the present clinical trial were promising and target the underlying pathology of RID, care must be taken in interpreting it, because of numerous limitations that we faced throughout the study. The first limitation was the small size of the studied subjects. Although we screened 130 patients for eligibility, patients’ cooperation was poor due to the COVID-19 pandemic. Second, we only examined the effects of one single concentration of this topical product, timolol 0.5% gel. It remains an area of research for future studies whether increasing the dose of the drug will be associated with higher efficacy without causing side effects or not. Third, regarding the stability of the formulation, for longer consumption time period, physicochemical as well as microbial quality control should be done. Finally, the study was not adjusted for other possible confounding factors including nutritional status, genetic, body mass index (BMI), and chemotherapy regimen, which could have potentially affected the occurrence and the intensity of dermatitis.

## Conclusion

This randomized controlled clinical trial showed that the preventive use of the timolol gel significantly delays and diminishes the maximum grade of ARD in breast cancer patients undergoing RT. Nevertheless, large multicenter randomized clinical trials (RCTs) are required to certify this novel concept for the prevention of ARD in breast cancer patients.

## Data Availability

All data generated or analyzed during this study are included in this published article.

## Abbreviations

RID: Radiotherapy induced dermatitis
QOL: Quality of life
ARD: Acute radiation dermatitis
SD-16: Skindex16
SD: Standard deviation
RTOG/EORTC: Radiation therapy oncology group and the european organization for research and treatment of cancer
CTCAE: Common terminology criteria for adverse events
RT: Radiotherapy
cAMP: Cyclic adenosine monophosphate
UVB: Ultraviolet b
API: Active pharmaceutical ingredient
MASCC: Multinational association of supportive care in cancer
KS: Kolmogorov–smirnov
IQR: Interquartile range
SPSS: Statistical package for social science
COVID-19: Coronavirus disease 2019
N: Number
HER2: Human epidermal growth factor Receptor 2
ER: Estrogen receptor
PR: Progesterone receptor
RCTs: Randomized clinical trial
